# Mid-Term Outcomes of a Pre-Cannulated Iliac Branched Device in the Treatment of Abdominal Aortoiliac Aneurysms: A Retrospective Analysis from a Single Center

**DOI:** 10.3390/jcm12196395

**Published:** 2023-10-07

**Authors:** Constantin Bonorden, Mohamed Shoura, Mateja Andic, Julia Kelley Hahn, Migdat Mustafi, Christian Schlensak, Mario Lescan

**Affiliations:** Department of Thoracic and Cardiovascular Surgery, University Medical Centre Tübingen, Hoppe-Seyler Strasse 3, 72076 Tübingen, Germany

**Keywords:** aorta, iliac, aneurysm, endovascular, hypogastric

## Abstract

The aim was to assess the mid-term results of the E-iliac branched device. Baseline and follow-up data of this monocentric retrospective cohort study including all consecutive patients with aortoiliac aneurysms treated with iliac branched devices between 2016 and 2023 were extracted from the hospital records. Preoperative and follow-up CT scans were analyzed regarding endoleaks, migration, aneurysm sac remodeling, and device patency. Overall, 50 devices were implanted in 38 patients with a median age of 69 (IQR 62–78) years, and 1.6 bridging stent grafts per vessel were implanted through transfemoral (22/50; 44%) or upper extremity access (28/50; 56%). Primary technical success and assisted technical success were 97% (37/38) and 100% (38/38), respectively. No migration, no type I or III endoleaks, no stroke, colonic ischemia, aneurysm rupture, or conversion during the early and mid-term follow-ups (11 months, IQR 5–26) were observed. Aneurysm sac enlargement or shrinkage was observed in 0% (0/38) and 16% (6/38) patients, respectively. E-iliac-related re-interventions were seen only during the early follow-up: two thrombectomies with bare-metal stent relining after thrombosis of the iliac limb. Bridging stent graft and E-iliac patency during the mid-term follow-up were 100%. E-iliac showed encouraging mid-term results in the treatment of aortoiliac aneurysms with high technical success and a low re-intervention rate.

## 1. Introduction

Concomitant iliac artery aneurysms are diagnosed in approximately 10% of the cases presenting with an abdominal aortic aneurysm (AAA), and their isolated occurrence is observed only in 2% of the diagnosed patients [1]. The elective repair is recommended at a diameter of ≥35 mm to prevent rupture [2]. In the past, open iliac aneurysm repair was the only available treatment option comprising the iliac aneurysm resection or ligation and the vascular reconstruction with conduits or the crossover bypass, respectively [3]. The evolution of endovascular therapy offered a new, less invasive treatment option consisting of the straight endovascular endograft and the coiling of the internal iliac artery (IIA) to prevent backflow to the aneurysm from this side branch [4]. Meanwhile, the endovascular repair of iliac artery pathologies has become the first-line therapy due to the lower invasivity and postoperative complication rate in comparison to open repair [2]. In this regard, Buck et al. demonstrated from the data of the National Inpatient Sample (including over 33,000 patients) a lower in-hospital mortality (1.8% vs. 0.5%; *p* < 0.001), a shorter hospital stay (7 d vs. 2 d; *p* < 0.001), and a reduced complication rate (18% vs. 7%; *p* < 0.001) for the endovascular repair of iliac aneurysms in comparison to the open repair [5]. Moreover, the development of iliac branched devices (IBD) with a directional branch to the IIA has facilitated the preservation of this artery with approved off-the-shelf treatment [6]. The current guidelines of the European Society for Vascular Surgery (ESVS) recommend preserving blood flow to at least one IIA to reduce postoperative buttock claudication, erectile dysfunction, and colonic ischemia [2]. Iliac branched devices were reported to improve outcomes in comparison to IIA embolization regarding buttock claudication, which was reported in up to 38% of the cases after IIA coverage [4].

The current indications include the iliac artery aneurysm repair, the re-interventions for type Ib endoleaks after endovascular aneurysm repair (EVAR) in the case of secondary dilatation of the distal landing zone in the common iliac artery (CIA) or already during the primary procedure as an alternative to the bell-bottom technique, which aims at distal sealing in an already dilated iliac artery with flared EVAR legs [7]. The wide applicability of IBD devices for the treatment of aortoiliac aneurysms has led to the availability of three approved off-the-shelf devices in Europe: the ZBIS (Cook Medical, Bloomington, IN, USA), the IBE (Gore, Flagstaff, AZ, USA), and the E-iliac (Artivion, Hechingen, Germany). The aim of this study was to report the mid-term results of the pre-cannulated E-iliac IBD device for the treatment of aortoiliac aneurysms in a real-world setting outlining its versatility for different indications.

## 2. Patients and Methods

### 2.1. Cohort Specifications

This retrospective cohort study included all consecutive patients with aortoiliac aneurysms treated between 2016 and 2023 with the E-iliac system, which has been our standard IBD device for the last 8 years. No other IBD devices have been used in our center during the study period. The procedures were isolated through the search of the default hospital database (SAP, Walldorf, Germany). The search criteria included the diagnosis (“aneurysm of the iliac artery”; ICD 10-“German modification” code: I72.3) and (in logical conjunction) the operation code for iliac branched devices (“operation and procedure keys” (OPS-) code: 5–38a.41). The study design and this manuscript were adapted to the STROBE guidelines for observational studies (see Supplementary Table S1).

### 2.2. Indications, Treatment Strategies, and Follow-up Structure

**Figure 1:**
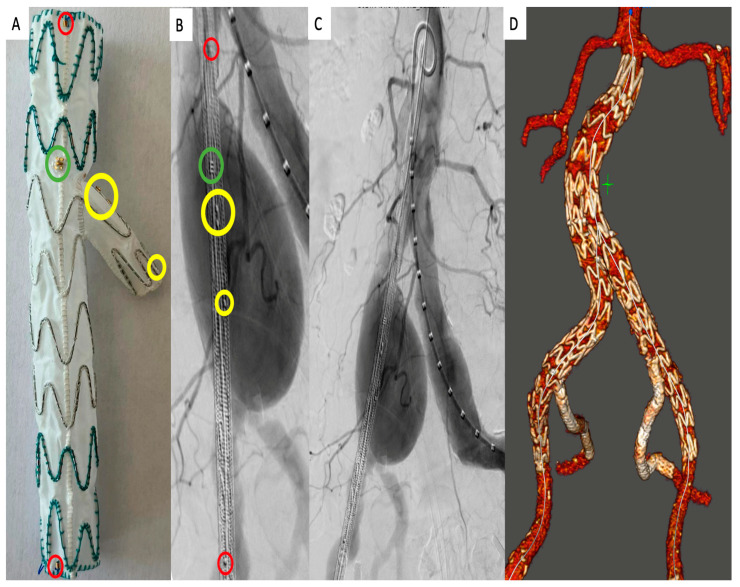
E-iliac branched device (IBD) design (**A**) ex situ and (**B**) in situ with the proximal and distal markers (red circle), the “E” orientation marker for the side branch (green circle), and the proximal and distal branch markers (yellow circle). (**C**) Implantation of an isolated E-iliac IBD in a patient with a common iliac artery aneurysm. (**D**) Postoperative 3D volume rendering of a patient with concomitant EVAR and bilateral E-iliac implantation.

At our center, iliac aneurysms and AAA have been treated from 2016 to 2018 at a maximum vessel diameter threshold of 30 mm and 50 mm, respectively. According to the change in the ESVS guidelines, the thresholds were adapted in 2019 to 35 mm and 55 mm, respectively [2]. The indications for IBD implantations (E-iliac, Artivion, Hechingen, Germany; Figure 1A–D) were the treatment of isolated iliac artery aneurysms (scenario 1), failing EVAR in the distal landing zone (in the presence of a progressive misalignment of the iliac limbs due to CIA dilatation during the follow-up with present or impending Type Ib endoleak; scenario 2) or EVAR with a preoperatively dilated distal landing zone > 25 mm or concomitant iliac aneurysms (scenario 3).

The isolated IBD implantation in scenario 1 (iliac aneurysms) was only performed if the proximal neck length in the native CIA exceeded 20 mm and the diameter of the proximal iliac artery was <17 mm to achieve an oversizing and sealing using the proximal device diameter. Otherwise, the proximal landing zone creation with an EVAR was necessary. 

All patients were followed up in our center according to the standard protocol including a patient interview, physical examination, and CT scan acquisition in the arterial and portal-venous phase with a slice thickness of 1 mm at 3, 6, and 12 months postoperatively and yearly thereafter. Moreover, the same CT scan protocol was used for the first postoperative CT scan prior to the discharge.

### 2.3. Operation Technique

After the percutaneous access or femoral cutdown to the common femoral artery, the E-iliac device was introduced to the iliac axis over a stiff guidewire (Lunderquist, Cook Medical, Bloomington, IN, USA) and a previously snared 18-inch pre-cannulation wire (V18, Boston Scientific, Marlborough, MA, USA). The pre-cannulation wire passes through the branch in a polyimide tube of the deployment system, whose proximal end lies within the CIA graft portion and its distal end is outside the EIA graft portion. The snaring was performed from the contralateral common femoral artery in case of the transfemoral approach or from the upper extremity access if previous EVAR implantation has been performed, and thus, a crossover maneuver was not feasible. The origin of the IIA and the aortic bifurcation was marked on the screen after the angiography. The device was then partially deployed until the full opening of the directional branch. An 8 Fr. Sheath (Destination, Terumo, Tokyo, Japan) was then introduced into the side branch over the 18-inch wire in the crossover technique. The IIA was cannulated, and a Rosenwire (Cook Medical, Bloomington, IN, USA) was placed in one of the IIA branches. The through-and-through wire was then removed, the 8 Fr. Sheath was positioned deeper in the IIA, and a bridging stent graft (E-ventus, Artivion, Hechingen, Germany or VBX, Gore, Flagstaff, AZ, USA) was then transferred through the sheath and deployed in the desired position. The VBX became available to our center in 2019 and was used in conjunction with the IBD if the landing zone of the bridging stent graft was deep in the IIA. For those cases, the 79 mm VBX length was used to avoid multiple E-ventus implantations. Due to the remote side branch diameter of 8 mm, proximal flaring in the branch was performed if the bridging stent graft diameter was <8 mm. The iliac branched device was then fully deployed, and proximal and distal landing zone apposition was supported via the compliant ballooning.

### 2.4. Data Acquisition, CT Analysis, and Outcome Definitions

All data were extracted from the medical records, intraoperative fluoroscopy, and CT imaging. CT scan analysis was performed with dedicated software (Therenva, Rennes, France), which allowed for the exact diameter (aneurysm diameters) and length (proximal landing zone length) measurements preoperatively, in the first week after the implantation and at the last follow-up CT scans. All diameters in this study were measured in the aortic centerline, which was automatically generated with the software after the placement of 5 “key markers” in the suprarenal aorta in both common femoral arteries and in both IIA. All diameter measurements were performed independently by two vascular surgeons. Initial measurements were accomplished by M.L. At a later time point, M.L. and M.S. performed another independent analysis of the CT scans in order to address the interobserver and intraobserver variability.

Type Ia, type Ib, type Ic, or type III endoleak presence; BSG and IBD patency; and the aneurysm sac diameter changes were assessed. Aneurysm sac shrinkage or enlargement was defined as aneurysm diameter reduction or increase ≥5 mm in the maximum sac diameter, respectively [8,9,10]. Component patency was defined as the absence of a thrombus in the endograft lumen or the absence of component stenosis ≥ 30% [9]. Primary technical success was characterized by the successful access and deployment of the IBD in the proximal and distal landing zones with the absence of type I or III endoleaks or stenosis ≥30% in the final angiography without unplanned additional procedures [9]. Type I endoleak was defined as the flow of the contrast agent in the aneurysm sac between the aortic (EVAR + IBD)/iliac artery wall (isolated IBD) and the endograft in the proximal (Ia) or distal (Ib) landing zones [9,11]. The contrast agent flow between the IIA wall and the bridging stent graft into the aneurysm sac was referred to as type Ic endoleak, whereas any detachment between the components, stent graft fracture, or detachment from the target vessels was defined as endoleak type III [11].

### 2.5. Statistical Analysis

The statistical analysis was performed with JMP^®^ 14 software (SAS, Cary, NC, USA). Categorical variables are presented as patient count (percentage), and continuous variables are reported as median (1st quartile; 3rd quartile) or as mean (±standard deviation) if the Kolmogorov–Smirnov test proved the normal distribution of the variable. Kaplan–Meier analysis with log-rank test was performed to compare the patency of the IBD components. Relative inter- and intraobserver variabilities of the diameter measurements were calculated including the individual standard deviation of the measurements as proposed by Popovic et al. [12]. *p* < 0.05 was considered significant.

## 3. Results

### 3.1. Patient Cohort and Procedural Parameters

The median age of the cohort was 69 (IQR 62–78; Table 1) years, 8% were women (3/38), and a median body mass index of 26 (IQR 24–30; Table 1) was calculated. Their cardiovascular risk factors are shown in Table 1, with hypertension (74% (28/38)) and nicotine abuse (39% (15/38)) being the most frequent.

A total of 38 patients were treated with 50 E-iliac IBD units of whom 10/50 (20%; Table 2) were implanted in a previous EVAR due to the distal sealing zone dilatation in the CIA. The previously implanted EVARs were Endurant (Medtronic, Santa Ana, CA, USA; 4/8), TREO (Terumo Aortic, Inchinnan, UK; 3/8), E-tegra (Artivion, Hechingen, Germany; 2/8), and Excluder (Gore, Flagstaff, AZ, USA; 1/8). Ten more IBDs (10/50; 20%) were implanted in patients with an untreated AAA and a CIA diameter > 25 mm.

Those pathologies were concomitantly treated with EVAR (E-tegra, Artivion, Hechingen, Germany) + IBD. Furthermore, 15 isolated iliac aneurysms had no adequate proximal IBD sealing zone length in the native CIA for the IBD and were treated with EVAR for the creation of the proximal sealing zone.

Overall, CIA aneurysms, IIA aneurysms, or combined CIA/IIA aneurysms were found in 36% (18/50), 8% (4/50), and 16% (8/50) of the pathologies, respectively.

The patient treatment consisted of EVAR + unilateral IBD, EVAR + bilateral IBD, unilateral isolated IBD, and bilateral isolated IBD in 16% (6/38), 24% (9/38), 55% (21/38), and 5% (2/38), respectively. Overall, 1.6 bridging stent grafts/vessels were implanted through transfemoral (44%, 22/50), brachial (34%; 17/50), or transaxillary (22%; 11/50) approach, of which 80% were E-ventus (40/50), VBX in 14% (7/50), or the combination of both in 6% (3/50). The transfemoral (crossover) approach resulted in a significant reduction in the intervention time in comparison to the upper extremity approach (160 min (IQR 116–217) vs. 246 min (IQR 172–319); *p* = 0.0107). The overall intervention time was 198 min (IQR 148–268), and the primary technical success was achieved in 97% (49/50). In one patient, an intraoperative EIA graft thrombosis was diagnosed during the implantation due to severe kinking of the EIA. Therefore, intraoperative thrombectomy with bare-metal stent relining of the EIA was performed. The assisted technical success was 100% (50/50).

### 3.2. Early Follow-Up Outcomes

At 30 days, one patient developed paraplegia on the first postoperative day after EVAR + uniiliac IBD. The patient received the treatment completion of a complex type 2 thoracoabdominal aneurysm repair after a previous TEVAR/BEVAR (6 weeks before). He died of a cardiac arrest on postoperative day 25. No further cases of mortality (1/38; 3%) or paraplegia (1/38; 3%) were observed (Table 3).

**Figure 2:**
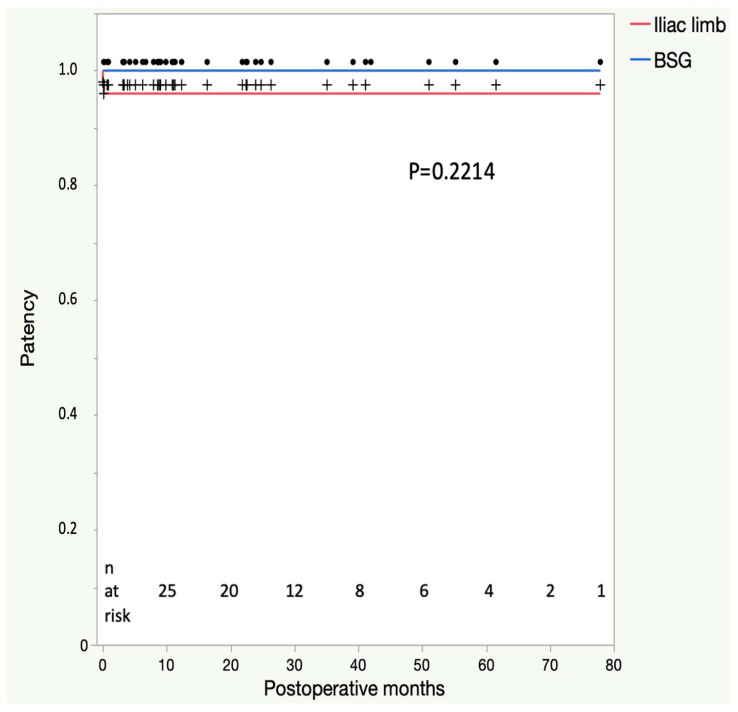
Comparison of the patency of the bridging stent graft (BSG) and the iliac limb component of the E-iliac device in the Kaplan–Meier analysis.

Furthermore, no cases of stroke, myocardial infarction, colonic ischemia, or wound complications needing surgical revision occurred within the first 30 days. There were two bleeding complications at the axillary access site needing surgical revision (2/38; 5%). The primary bridging stent graft and the primary IBD patency were 100% and 94% (Figure 2), respectively, with one already described with intraoperative EIA thrombosis and another at postoperative day 4, which was treated with thrombectomy and PTA and remained patent throughout the follow-up. Type II endoleaks were observed in 16/38 patients (48%). Type I and III endoleaks were not found in the first postoperative CT scan before discharge.

### 3.3. Mid-Term Follow-Up Outcomes

During the median follow-up of 11 (IQR 5–26) months, two more patients died (2/37; 5%): one from lung cancer at 14 months, and another patient with an infected TEVAR at 51 months (Table 4).

No stroke, myocardial infarction, paraplegia, colonic ischemia aneurysm rupture, or conversion to open aneurysm repair was observed. Furthermore, buttock claudication and re-interventions were not detected during the follow-up. No new type I or III endoleaks occurred, whereas type 2 endoleaks showed a high regression rate of 63% (*p* = 0.0099) during the follow-up. Sac enlargement has not been observed. The aneurysm sac shrinkage rate ≥ 5 mm occurred in 16% of the cohort (6/37), whereas sac stability was present in 84% (31/37; Table 4). The patency of the BSG and the IBD was 100% in the mid-term follow-up.

The relative intraobserver variability difference for diameter measurements in the centerline was 0.07% with an individual standard deviation of 1.2%. The relative interobserver variability difference was 0.04% with an individual standard deviation of 0.7%.

## 4. Discussion

### 4.1. Status of the IBD in the Treatment of Aortoiliac Aneurysms

Previous studies have emphasized the importance of the preservation of at least one iliac intern aneurysm during open or endovascular surgery to prevent postoperative complications [13]. Jean Baptiste et al. reported a 3% rate of fatal pelvic ischemia in 71 patients after IIA occlusion in EVAR and the occurrence of buttock claudication in 25%, particularly among younger patients [14]. Bosanquet et al. showed a high rate of buttock claudication and erectile dysfunction in their meta-analysis including IIA coverage following EVAR [13]. Concurrently, the repair of iliac aneurysms with IBDs was reported to significantly reduce the occurrence of buttock claudication in comparison to the IIA occlusion: Taudorf et al. showed a 38% claudication rate in patients with IIA occlusion in comparison to no claudication in the IBD group [4], which was comparable to our study with no buttock claudication at the mid-term follow-up. Currently, the results of the most frequently used devices have been reported. In a prospective multicenter trial including IBE (Gore, Flagstaff, AZ, USA) with concomitant AAA repair, a 95% patency of the iliac limb, absence of type I and III endoleaks, and freedom from secondary intervention of >90% [15,16] were shown. A propensity-score-matched comparison of IBE with the ZBIS (Cook Medical, Bloomington, IN, USA) device showed comparable mid-term results with 100% device patency, 93 (IBE)/97% (ZBIS) freedom from re-intervention and 87% (IBE)/97% (ZBIS) freedom from type I and type III endoleaks [17]. Those data are comparable to the results in our study, which showed considerable mid-term results (median follow-up of 12 months) of the E-iliac device with primary patency of the bridging stent graft and secondary iliac limb patency of 100%, freedom from type I and type III endoleaks, and from any secondary re-intervention in the >30 d period. Regarding anatomical feasibility, a study comparing the instructions for use criteria of all three off-the-shelf IBD devices showed a significantly higher feasibility rate with the ZBIS (53%) in comparison to IBE (34%) and E-iliac (27%) [18]. Thereby, the major limitation of E-iliac applicability was the offspring angle of the AII exceeding 50°. However, in the real-world setting, as in our study, more liberal criteria for the IBD treatment choice are applied to subject patients with severe comorbidities to a less invasive procedure. Thus, the authors reported that the overall feasibility of their study increased from 66% to 96% after the use of looser feasibility criteria for all three studied devices [18].

### 4.2. Results of the E-iliac IBD Studies

Prior to our study, Mylonas et al. reported a technical success of 100% for E-iliac and freedom from type Ia endoleaks of 87% at 1 year. During one year of the study period, two CIAs and two EIAs thrombosed, thereof three thromboses appeared after 5–6 months [6]. Two thromboses of the EIA were also observed in our study; however, both were at an early stage during the hospital stay and both remained patent after the early re-intervention. In both cases, the kinking of the EIA was identified as the reason for the thrombosis, which was fixed with the relining of the iliac limb (with PTA or bare-metal stent implantation). Brunkwall et al. reported one-year results of 48 E-iliac devices and also found edge stenosis and thrombosis in two EIAs, which needed re-intervention [19]. Thus, the extern iliac artery limb of the E-iliac needs to be carefully evaluated in the completion angiography for kinking and stenosis, particularly in tortuous anatomies, as it appears to be the major complication site of this system.

The ABRAHAM study reported the treatment of 18 IIA aneurysms with the E-iliac system and showed a considerable rate of re-interventions due to one EIA occlusion, two type IB endoleaks, and two type IIIB endoleaks. This indicates a considerably higher endoleak and re-intervention rate than in the present study, where type I and III endoleaks were not observed [20]. The treatment of the IIA aneurysms is not mentioned in isolation as an indication in the instructions of use of E-iliac; therefore, the treatment of this pathology is an off-label use. However, as Dueppers et al. stated, the treatment of IIA aneurysms is technically challenging but feasible with good early and mid-term results [20]. In our study, four patients with isolated IIA aneurysms were successfully treated and had no endoleaks or re-interventions during the follow-up.

Recently, Yazar et al. analyzed the compatibility of the E-iliac with Endurant II/IIs (Medtronic, Santa Rosa, CA, USA) EVAR for concomitant treatment of AAA and iliac artery aneurysms, the creation of distal EVAR landing zone in dilated CIA, or in absent proximal landing zone for the IBD [21]. The authors reported a technical success of 100%, and only one E-iliac-related re-intervention in one year, which is comparable to our results with two re-interventions at a median follow-up of 11 (IQR 5–26) months [21]. In our study, 15 patients (24 IBD) were treated with concomitant EVAR, however, with the E-tegra EVAR device in order to use the same manufacturer with approved compatibility. Nevertheless, 10 E-iliac devices in eight patients were successfully implanted for the treatment of type Ib endoleaks after previous EVAR with four different devices (E-tegra, Endurant, Treo, Excluder).

In summary, E-iliac showed a high versatility regarding the indications (Table 2), implantation sequence, and compatibility with different EVAR devices during the endovascular re-do procedures.

The main limitation of this study is the retrospective design with an overall small number of included patients. The patient number is, however, comparable to other studies published about this specific IBD device, and with the growing popularity of meta-analyses, our data may play an important role in the evaluation of future results, e.g., for the comparison of the results of different IBD devices. Nevertheless, the power of this retrospective analysis including only 36 patients may be limited in drawing any final conclusion about the safety and durability of the E-iliac system. Regarding durability, the median follow-up of 11 (IQR 5–26) months is a rather short mid-term follow-up period, which may lead to an underestimation of the follow-up complications, particularly in terms of E-iliac/bridging stent graft patency and material fatigue. Thus, the Cox regression analysis offers some insights into the patency of this IBD but may be biased by the small cohort and the incomplete follow-up at one year, which is another limitation of retrospective studies. Comparisons of different manufacturers are important to ensure that the patients receive a durable device that is most suitable for their anatomical predisposition, and optimally, within the instructions for use of the respective manufacturer. Furthermore, single-center retrospective data are prone to bias, although standardized techniques are employed during the evaluation. Another limitation is the use of only one IBD type in our center, thus a comparison with other devices was not feasible. Future comparative studies of different endograft types may discover the advantages of certain anatomical preconditions, which were not possible to assess in our study.

## 5. Conclusions

E-iliac showed encouraging mid-term results and high versatility in the treatment of different types of aortoiliac aneurysms with high technical success and low re-intervention rates. The importance of perfect alignment of the EIA graft portion to the vessel wall, assisted with additional ballooning or bare-metal stenting, must be mentioned to avoid EIA graft thrombosis.

## Figures and Tables

**Table 1:** Demographic data and co-morbidities.

Parameter	Overall
n	38 (100)
Age (year; n (%))	69 (IQR 62–78)
Sex (female; n (%))	3 (8)
Body mass index	26 (IQR 24–30)
Hypertension, n (%)	28 (74)
Nicotine Abuse, n (%)	15 (39)
Diabetes, n (%)	5 (13)
Dyslipoproteinemia, n (%)	7 (18)
COPD, n (%)	6 (16)
Previous CABG, n (%)	3 (8)
Atrial fibrillation, n (%)	8 (21)
Previous stroke, n (%)	5 (13)
Peripheral artery disease, n (%)	5 (13)
Glomerular filtration rate, n (%)	
CKD Stage I (GFR: ≥90 mL/min)	14 (37)
CKD Stage II (GFR: 60–89 mL/min)	18 (47)
CKD Stage III (GFR: 30–59 mL/min)	5 (13)
CKD Stage IV (GFR: 15–29 mL/min)	0 (0)
CKD Stage V (GFR: <15 mL/min)	1 (3)
Previous abdominal surgery, n (%)	17 (45)

IQR, interquartile range; COPD, chronic obstructive pulmonary disease; CABG, coronary artery bypass graft; CKD, chronic kidney disease; GFR, glomerular filtration rate.

**Table 2:** Indications, anatomical and procedural data.

Parameter	Overall
Implanted IBD devices, n (%)	50 (100)
Indications (n = 50; IBD units)	
Previous EVAR with DLZ dilation/endoleak, n (%)	10 (20)
AAA treatment with DLZ absence, n (%)	10 (20)
CIA aneurysm, n (%)	18 (36)
IIA aneurysm, n (%)	4 (8)
Combined CIA/IIA aneurysm, n (%)	8 (16)
Iliac bifurcation diameter (mm)	25 (IQR 20–32)
BSG access (n = 50; IBD units)	
Transfemoral, n (%)	22 (44)
Brachial, n (%)	17 (34)
Transaxillary, n (%)	11 (22)
Procedure description (n = 38; patients)	
EVAR + unilateral IBD, n (%)	6 (16)
EVAR + bilateral IBD, n (%)	9 (24)
Unilateral isolated IBD, n (%)	21 (55)
Bilateral isolated IBD, n (%)	2 (5)
Bridging stent grafts per vessel	1.6
Bridging stent graft type (n = 50; IBD units)	
E-ventus, n (%)	40 (80)
VBX, n (%)	7 (14)
Combined, n (%)	3 (6)
Operation time (min)	197 (IQR 136–262)
Primary technical success (n = 38; patients), n (%)	37 (97)
Assisted technical success (n = 38; patients), n (%)	38 (100)
Intraoperative endoleaks (n = 38; patients), n (%)	
Type I	0 (0)
Type III	0 (0)
Unplanned adjunctive procedures (n = 38; patients), n (%)	1 (3)

EVAR, endovascular aneurysm repair; DLZ, distal landing zone; CIA, commune iliac artery; IIA, intern iliac artery; IQR, interquartile range; BSG, bridging stent graft; IBD, iliac branched device.

**Table 3:** Early clinical outcomes.

Parameter	Overall
n (%)	38 (100)
ICU stay	0 (0)
Stroke, n (%)	0 (0)
Mortality, n (%)	1 (3)
Myocardial infarction, n (%)	0 (0)
Paraplegia, n (%)	1 (3)
Colonic ischemia, n (%)	0 (0)
Bleeding complications needing revision, n (%)	2 (5)
Wound complications needing revision, n (%)	0 (0)
Endoleaks before discharge (patients)	
Type I, n (%)	0 (0)
Type II, n (%)	16 (43)
Type III, n (%)	0 (0)

ICU, intensive care unit; BSG, bridging stent graft; IBD, iliac branched device.

**Table 4:** Mid-term clinical outcomes (>30 days).

Parameter	Overall
n (%)	37 (100)
Follow-up length (months)	11 (IQR 5–26)
Stroke, n (%)	0 (0)
Mortality, n (%)	2 (5)
Myocardial infarction, n (%)	0 (0)
Paraplegia, n (%)	0 (0)
Colonic ischemia, n (%)	0 (0)
Aneurysm rupture, n (%)	0 (0)
Buttock claudication, n (%)	0 (0)
Conversion to open repair, n (%)	0 (0)
Re-interventions	0 (0)
Endoleaks at last follow-up (patients)	
Type I, n (%)	0 (0)
Type II, n (%)	6 (16)
Type III, n (%)	0 (0)
Type II endoleak resolution, n (%)	−10 (−63)
Aneurysm growth	
Sac enlargement, n (%)	0 (0)
Sac stability, n (%)	31 (84)
Sac shrinkage, n (%)	6 (16)

IQR, interquartile range; BSG, bridging stent graft; IBD, iliac branched device.

## Data Availability

The data underlying this article will be shared upon reasonable request from the corresponding author.

## Supplementary Materials

The following supporting information can be downloaded at: https://www.mdpi.com/article/10.3390/jcm12196395/s1, Table S1: STROBE Statement—Checklist of items that should be included in reports of cohort studies.
